# Vibrio parahaemolyticus Senses Intracellular K^+^ To Translocate Type III Secretion System 2 Effectors Effectively

**DOI:** 10.1128/mBio.01366-18

**Published:** 2018-07-24

**Authors:** Sarunporn Tandhavanant, Shigeaki Matsuda, Hirotaka Hiyoshi, Tetsuya Iida, Toshio Kodama

**Affiliations:** a Department of Bacterial Infections, Research Institute for Microbial Diseases, Osaka University, Osaka, Japan; b Department of Medical Microbiology and Immunology, School of Medicine, University of California, Davis, Davis, California, USA; Institut Pasteur

**Keywords:** T3SS, *Vibrio parahaemolyticus*, effector, gatekeeper, translocator

## Abstract

Many Gram-negative bacterial symbionts and pathogens employ a type III secretion system (T3SS) to live in contact with eukaryotic cells. Because T3SSs inject bacterial proteins (effectors) directly into host cells, the switching of secretory substrates between translocators and effectors in response to host cell attachment is a crucial step for the effective delivery of effectors. Here, we show that the protein secretion switch of Vibrio parahaemolyticus T3SS2, which is a main contributor to the enteropathogenicity of a food poisoning bacterium, is regulated by two gatekeeper proteins, VgpA and VgpB. In the absence of these gatekeepers, effector secretion was activated, but translocator secretion was abolished, causing the loss of virulence. We found that the K^+^ concentration, which is high inside the host cell but low outside, is a key factor for VgpA- and VgpB-mediated secretion switching. Exposure of wild-type bacteria to K^+^ ions provoked both gatekeeper and effector secretions but reduced the level of secretion of translocators. The secretion protein profile of wild-type bacteria cultured with 0.1 M KCl was similar to that of gatekeeper mutants. Furthermore, depletion of K^+^ ions in host cells diminished the efficiency of T3SS2 effector translocation. Thus, T3SS2 senses the high intracellular concentration of K^+^ of the host cell so that T3SS2 effectors can be effectively injected.

## IMPORTANCE

The pathogenesis of many Gram-negative bacterial pathogens arises from a type III secretion system (T3SS), whereby bacterial proteins (effectors) are directly injected into host cells. The injected effectors then modify host cell functions. For effective delivery of effector proteins, bacteria need to both recognize host cell attachment and switch the type of secreted proteins. Here, we identified gatekeeper proteins that play important roles in a T3SS2 secretion switch of Vibrio parahaemolyticus, a causative agent of food-borne gastroenteritis. We also found that K^+^, which is present in high concentrations inside the host cell but in low concentrations outside, is a key factor for the secretion switch. Thus, V. parahaemolyticus senses the high intracellular K^+^ concentration, triggering the effective injection of effectors.

## INTRODUCTION

Many Gram-negative bacterial symbionts and pathogens utilize a type III secretion system (T3SS) for their benefit and/or pathogenesis. The T3SS is a sophisticated secretion system for direct delivery of effectors into the host cell cytosol. For the efficient translocation of effectors, T3SS substrate secretion is generally divided into three phases, which are regulated by specific components in a specific order (1). First, extracellular secretion of needle protein (the early substrate) leads to the formation of tube-like structures. When the needle of a bacterium reaches an appropriate length, molecular ruler proteins switch secretion to the second phase. Translocators are the middle substrates: they localize at the tip of the T3SS needle and form a pore in the host plasma membrane to create a pathway for the effectors. Bacteria promote to secrete the late substrates, effectors, after the organism is in contact with the host cells to achieve the most efficient translocation. The switching of T3SS secretion from the middle (translocators) to the late substrates (effectors) is controlled by a “gatekeeper” protein. Functional knockout of gatekeeper genes disrupts orderly T3SS secretion and causes hypersecretion of effectors (2–10). Blockage of effector secretion by the gatekeeper is released upon exposure to specific stimulators that reflect the host intracellular milieu, such as low calcium concentrations and pH shifts (9, 11). Although several models for host cell sensing have been proposed, the exact mechanism of substrate switching by gatekeepers remains unknown.

Vibrio parahaemolyticus is a causative agent of food poisoning worldwide (12–15). Most clinical isolates from patients with diarrhea possess two sets of the T3SS gene, one set on each chromosome (16, 17). A number of reports indicate that T3SS2, which is encoded by a gene(s) on an 80-kb V. parahaemolyticus pathogenicity island (Vp-PAI), is essential for enterotoxicity in animal infection models (18, 19). Several T3SS2 effectors, including the effector responsible for enterotoxicity, have been identified (20–24). T3SS2 is therefore considered to be a major virulence factor for enteropathogenicity of this bacterium. However, the precise mechanism of action of T3SS2 remains unknown, because there are many functionally uncharacterized genes on Vp-PAI. In this study, we have identified two hypothetical genes in Vp-PAI as genes encoding gatekeeper proteins of T3SS2. The deletion of these genes activates T3SS2 effector secretion but diminishes the secretion of translocators. In addition, we have found that a high intracellular concentration of K^+^ ions is a host factor that switches T3SS2 secretion from the middle to late substrates. Thus, V. parahaemolyticus T3SS2 recognizes the host cell attachment by sensing high intracellular concentrations of K^+^ ions, which enables effective translocation of T3SS2 effectors.

## RESULTS

### Identification of two hypothetical genes encoded on Vp-PAI (*vpa1360* and *vpa1359*) that are essential for V. parahaemolyticus T3SS2-dependent biological activities.

**FIG 1:**
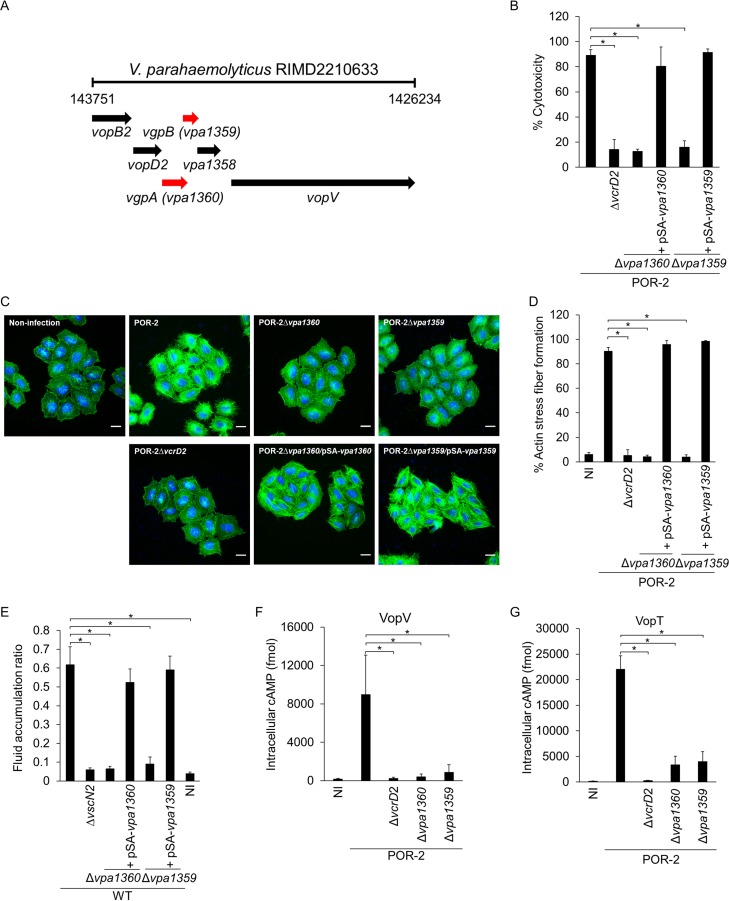
Identification of two Vp-PAI-encoded hypothetical genes (*vpa1360* and *vpa1359*) that are essential for translocation of V. parahaemolyticus T3SS2 effectors. (A) Organization of *vpa1360* and *vpa1359* genes in the Vp-PAI region on chromosome 2 of V. parahaemolyticus RIMD2210633. (B) *vpa1360* and *vpa1359* are required for T3SS2-dependent cytotoxicity against Caco-2 cells after 6 h of infection. The bars show the means from three independent experiments. Error bars indicate standard deviations (SDs). Values that are significantly different (*P* ≤ 0.05) are indicated by a bar and asterisk. (C) T3SS2-dependent actin stress fiber formation. Staining of F-actin (green) and host cell and bacterial DNA (blue) in HeLa cells after their infection with isogenic V. parahaemolyticus mutant strains at an MOI of 100 for 3 h. Bars, 100 µm. (D) Percentage of cells exhibiting actin stress fibers after infection with a *vpa1360* or *vpa1359* deletion mutant and their complementary strains. One hundred cells from several fields were analyzed by microscopy in each experiment to determine whether stress fiber formation was induced. The means plus SDs from three independent experiments are presented. The asterisks indicate statistically significant differences (*, *P* ≤ 0.05). NI, noninfected control. (E) *vpa1360* and *vpa1359* are required for T3SS2-dependent enterotoxicity in the rabbit ileal loop model. The fluid accumulation ratio represents the volume of fluid (in milliliters) per length of intestine (in centimeters) after 15 h of infection. The bars represent the means from two independent experiments. Error bars indicate SDs. *, *P* ≤ 0.05. (F and G) Effects of *vpa1360* and *vpa1359* gene deletion on translocation of T3SS2 effector (VopV and VopT), assessed by CyaA-based translocation assay. Intracellular cAMP level in Caco-2 cells infected with VopV- or VopT-CyaA-expressing isogenic V. parahaemolyticus strains was determined after 1.5 h of infection. The bars present the means from three independent experiments. Error bars indicate SDs. *, *P* ≤ 0.05.

Our functional analysis of genes encoded on Vp-PAI identified two hypothetical genes (*vpa1360* and *vpa1359*) that have a critical role in T3SS2-dependent biological activities. *vpa1360* and *vpa1359* are arranged in tandem with 89 bp overlapping and are encoded downstream of the genes for T3SS2 translocators, *vopB2* and *vopD2* (Fig. 1A) (16). Although VPA1360 and VPA1359 proteins have no homology to protein sequences in the database with known functions, the levels of expression of these genes are reportedly upregulated not only by bile, which is a strong inducer of Vp-PAI genes *in vitro*, but also during intestinal colonization of an infant rabbit, an oral infection model *in vivo* (25, 26). In addition, a recent genetic analysis revealed that *vpa1360* was identified as a V. parahaemolyticus gene required for viability and colonization in the distal small intestine of an infant rabbit (25). These reports imply that *vpa1360* and *vpa1359* are indispensable for the enteropathogenicity of this bacterium. We therefore constructed *vpa1360* and *vpa1359* deletion mutants from wild-type strain RIMD2210633 (WT), POR-2 (WT Δ*tdhAS* Δ*vcrD1*; a thermostable direct hemolysin [TDH]- and T3SS1 export apparatus-deficient strain), and POR-2Δ*vcrD2* (WT Δ*tdhAS* Δ*vcrD1* Δ*vcrD2*; a TDH- and T3SS1 and T3SS2 export apparatus-deficient strain). We first confirmed that the deletion of each gene did not affect the joint protein production in several background strains (see Fig. S1A, B, and C in the supplemental material). We then examined the phenotypic characteristics of deletion mutants and the T3SS2-dependent biological activities, including cytotoxicity (Fig. 1B), actin stress fiber formation (Fig. 1C and D), and enterotoxicity (Fig. 1E). V. parahaemolyticus contains several virulence factors such as TDH, T3SS1, and T3SS2 that are responsible for cytotoxicity (19). However, the cytotoxicity toward Caco-2 cells is a characteristic effect of T3SS2 *in vitro*, and an ADP-ribosyltransferase effector, VopT, is partly involved in this cytotoxicity (22). The induction of actin stress fiber formation is a coordinated effect of three T3SS2 effectors, VopC, VopL, and VopO (17, 20, 21, 24, 27). To avoid the cytotoxic effects of TDH and T3SS1, we therefore used the POR-2 strain as a parental strain for the *in vitro* infection assay to focus on the T3SS2-dependent effect. It is clearly demonstrated that T3SS2 is the main contributor of the induction of fluid accumulation in rabbit ileal loop models, an *in vivo* animal model of diarrhea (18, 19). The T3SS2 effector VopV is responsible for F-actin binding which required for V. parahaemolyticus enterotoxicity (23, 28). We therefore assessed the enterotoxicity using WT and derivative strains. As a result, all T3SS2-dependent biological activities of *vpa1360* and *vpa1359* deletion mutants were almost identical to those of T3SS2-deficient strains (e.g., POR-2Δ*vcrD2* or WTΔ*vscN2*). The T3SS2-dependent biological activities were restored by in *trans* complementation with the *vpa1360* or *vpa1359* gene.

10.1128/mBio.01366-18.2 FIG S1  No polar effect from deletion of *vpa1360* and *vpa1359*. Download FIG S1, TIF file, 0.4 MB. Copyright © 2018 Tandhavanant et al. 2018 Tandhavanant et al. This content is distributed under the terms of the Creative Commons Attribution 4.0 International license.

Because the translocation of effectors into host cells is the main function of T3SS, we then determined the effect of *vpa1360* or *vpa1359* deletion on the translocation of T3SS2 effectors by using CyaA-based translocation assays. The levels of translocated T3SS2 effectors (VopV and VopT) were significantly lower in POR-2Δ*vpa1360-* or POR-2Δ*vpa1359*-infected cells than the levels in cells infected with POR-2 (Fig. 1F and G). However, there were no significant differences in cytotoxicity among strains under these conditions (see Table S1 in the supplemental material), indicating the possibility that gene deletions cause a malfunction of T3SS2. These results suggested that *vpa1360* and *vpa1359* are essential elements for exertion of T3SS2-dependent biological activities.

10.1128/mBio.01366-18.7 TABLE S1  Cytotoxicity against Caco-2 cells by V. parahaemolyticus (POR-2) and derivative strain after 1.5- or 3-h infection. Download TABLE S1, DOCX file, 0.01 MB. Copyright © 2018 Tandhavanant et al. 2018 Tandhavanant et al. This content is distributed under the terms of the Creative Commons Attribution 4.0 International license.

### Deletion of *vpa1360* (*vgpA*) and *vpa1359* (*vgpB*) genes causes an oversecretion of T3SS2 effectors but diminishes the secretion of translocators.

**FIG 2:**
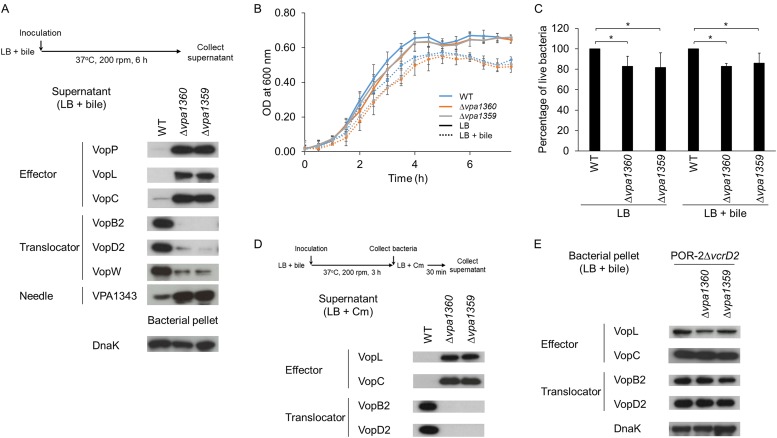
Deletion of *vpa1360* (*vgpA*) and *vpa1359* (*vgpB*) genes causes oversecretion of T3SS2 effectors but diminishes secretion of translocators. (A) Effects of *vpa1360* (*vgpA*) and *vpa1359* (*vgpB*) gene deletions on secretion of T3SS2 proteins. Immunoblotting of T3SS2-secreted proteins (effectors, translocators, and needle) from the supernatant of cultures grown in LB broth for 6 h. DnaK was used as a loading control. (B) Growth curve of *vpa1360* and *vpa1359* mutants and parental strains in LB or LB containing crude bile. The bacteria were cultured at 37°C, and the optical density (OD) was measured at 600 nm every 30 min. The data points are the means from three independent experiments. The error bars represent SDs. (C) Analysis of percentage of live V. parahaemolyticus and derivative strains. The bacterial suspensions from culture in LB or LB containing crude bile for 6 h were stained using the LIVE/DEAD BacLight bacterial viability kit (Molecular Probe). The percentage of live bacteria for the derivative strains was compared to that for the parental strain. The heat-killed bacteria were used as 100% dead bacteria. The bars are the means from three independent experiments. Error bars indicate SDs. *, *P* ≤ 0.05. (D) New protein synthesis is not needed to switch the secretion protein profile of T3SS2 observed in *vpa1360* and *vpa1359* deletion mutants. After 3 h of incubation in the presence of 0.04% bile, bacteria were harvested and resuspended in LB broth containing chloramphenicol (Cm) to inhibit protein synthesis. After 30 min of incubation, the supernatants were analyzed by immunoblotting. (E) Effect of *vpa1360* and *vpa1359* deletion on total production of T3SS2-related proteins in T3SS2-deficient strains. The bacterial pellet samples from indicated strains were analyzed by immunoblotting to determine whether deletion of *vpa1360* or *vpa1359* affects the production of T3SS2-related proteins. DnaK was used as a loading control.

To determine the cause of the malfunction of T3SS2 by deleting *vpa1360* or *vpa1359*, immunoblotting experiments were conducted to examine the effect of the gene deletion on secretion of T3SS2-related proteins in the presence of crude bile, a potent inducer of T3SS2 (Fig. 2A) (26). Interestingly, the levels of secretion of T3SS2 effectors (VopP, VopL, and VopC) and needle filament (VPA1343) increased in Δ*vpa1360* and Δ*vpa1359* mutants compared with those of WT. In contrast, the levels of secretion of translocators (VopB2, VopD2, and VopW) decreased in *vpa1360* or *vpa1359* gene deletion mutant. Deletion of these genes did not affect bacterial growth (Fig. 2B), but the percentage of live Δ*vpa1360* and Δ*vpa1359* mutants were slightly decreased (Fig. 2C). These results indicate the possibility that membrane damage from dead bacteria leads to the hypersecretion phenotype. Therefore, we confirmed these phenotypes from bacteria cultured in Dulbecco’s modified Eagle’s medium (DMEM). The gene deletion did not affect either the growth rate or the percentage of live bacteria (Fig. S2A and B). Silver staining and immunoblotting of culture supernatant samples from DMEM revealed that both POR-2Δ*vpa1360* and POR-2Δ*vpa1359* strains promoted secretion of all the T3SS2-related proteins except for VopB2 (Fig. S2C and D). These results suggest that changes in the levels of T3SS2 secretion by *vpa1360* and *vpa1359* deletion mutants are not caused by passive diffusion from membrane damage of dead bacteria.

10.1128/mBio.01366-18.3 FIG S2  *vpa1360* and *vpa1359* mutants exhibit altered patterns of T3SS2 secretion from the pattern of the parental strain without differences in bacterial growth or number of bacteria with damaged membrane. Download FIG S2, TIF file, 1.8 MB. Copyright © 2018 Tandhavanant et al. 2018 Tandhavanant et al. This content is distributed under the terms of the Creative Commons Attribution 4.0 International license.

To determine whether newly synthesized protein is needed for the T3SS2 secretion switch, we collected the supernatants after the bacteria were exposed to chloramphenicol, a protein synthesis inhibitor, and examined them by immunoblotting. Chloramphenicol treatment did not strongly affect the secretion phenotype of T3SS2 (Fig. 2D).

We next determined whether deletion of these genes affects the T3SS2-related protein production. We used strain POR-2Δ*vcrD2* (null T3SS2 secretion) as a parental strain, because this strain allows us to determine the total production levels of T3SS2-related proteins by comparing the intensity of protein bands from bacterial pellet samples. *vpa1360* and *vpa1359* deletion mutants that were derived from the POR-2Δ*vcrD2* strain produced T3SS2 effectors (VopL and VopC) and T3SS2 translocators (VopB2 and VopD2) in the bacterial pellet at levels equivalent to those of the parent strain (Fig. 2E). These results collectively suggested that the deletion of *vpa1360* or *vpa1359* causes a drastic change in the profiles of T3SS2-secreted proteins without changing the levels of production of T3SS2-related proteins.

It is proposed that protein secretion through the injectisome of T3SS occurs in consecutive steps and that the presence of a variety of switching mechanisms ensures the secretion hierarchy. Translocators (middle substrates) are abundantly secreted in culture supernatants under *in vitro* culture conditions rather than effectors (late substrates), and the transition of the protein secretion from middle to late substrates subsequently occurred when the host cells were in contact with the bacteria (29). The protein that causes the switch from middle- to late-substrate secretion is termed the gatekeeper (29). In the absence of gatekeeper protein, secretion of translocators is attenuated, and effectors are oversecreted through their T3SS (6, 11, 30). The phenotypic characteristics of gatekeeper knockout strains of other pathogenic bacteria were similar to those observed in Δ*vpa1360* and Δ*vpa1359* mutants. We therefore designated the VPA1360 and VPA1359 proteins as VgpA (Vibrio parahaemolyticus gatekeeper protein A) and VgpB, respectively, and their possible role as gatekeepers was examined in the following experiments.

### VgpA is translocated into host cells via T3SS2.

**FIG 3:**
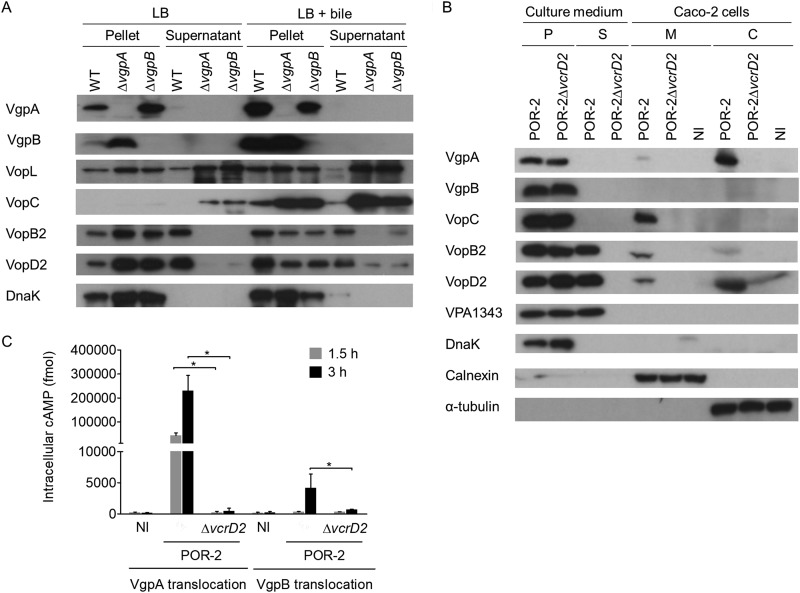
VgpA is not secreted into culture supernatant but translocated into host cells. (A) VgpA and VgpB are not detected in supernatants, even when the bacteria are cultured in the presence of crude bile. Bacterial pellets and supernatants from V. parahaemolyticus strains cultured in LB broth with or without 0.04% crude bile were probed for T3SS2-related proteins. (B) T3SS2-dependent translocation of VgpA, but not VgpB, into host cells. Cultures of Caco-2 cells that had been infected with V. parahaemolyticus strains for 3 h were separated into their culture medium and infected cells. The culture media were separated into a bacterial pellet (P) and supernatant (S). The infected cells were fractionated into host membrane (M) and cytosol (C) samples. Each fraction was probed for T3SS2-related proteins (VgpA, VgpB, VopC, VopB2, VopD2, and VPA1343). DnaK, calnexin, and tubulin were used as markers of bacteria, host membranes, and the host cytosol, respectively. NI, noninfected control. (C) Translocation of VgpA and VgpB into host cells evaluated by CyaA-based translocation assay. Intracellular cAMP levels reflect the translocation of VgpA-CyaA or VgpB-CyaA after 1.5 or 3 h of infection. The bars are the means from three independent experiments. Error bars indicate SDs. *, *P* ≤ 0.05.

Several gatekeepers are localized either in the bacterial membrane or in the cytosol (11, 31, 32), and they are secreted upon exposure to conditions that mimic host cell contact (7, 8). Immunoblot analysis of samples from V. parahaemolyticus in the absence of host cells revealed that neither VgpA nor VgpB was detected in culture supernatant, even in the presence of bile (Fig. 3A). However, we found that VgpA, but not VgpB, was detected in the cytosol fraction of host cells (Fig. 3B). We used the POR-2 strain to eliminate the cytotoxic effect of T3SS1 and TDH. The translocation of VgpA into the host cell was observed in cells infected with strain POR-2, but not POR-2Δ*vcrD2*. This T3SS2-dependent translocation of VgpA into host cells was also confirmed with a CyaA-based translocation assay (Fig. 3C). The intracellular cyclic AMP (cAMP) level was significantly higher only in cells infected with the CyaA-fused VgpA-expressing POR-2 strain. In contrast, although the intracellular cAMP level was slightly elevated after 3 h of infection, the translocation of VgpB-CyaA was lower than that of VgpA-CyaA (Fig. 3C). The low efficiency of VgpB translocation (Fig. 3C) may cause the absence of VgpB in the fractionation of infected host cells (Fig. 3B). These results indicated that T3SS2-dependent VgpA and VgpB secretion might occur only upon contact with host cells, and they also imply the existence of a host cell-derived factor that facilitates secretion of these proteins.

### Identification of K^+^ as a factor that shifts the T3SS2 secretion from middle to late substrates.

We then wanted to identify host cell factors that specifically shift T3SS2 secretion from middle to late substrates. Several conditions, including a low concentration of calcium, pH shifts, and Congo red, have been proposed as factors that cause transition of the T3SS substrate secretion (11, 31, 33). Protein secretion from T3SS1, the T3SS gene cluster which is similar to that of *Yersinia* spp. (34), was blocked in the presence of calcium. In contrast, the addition of calcium to the medium did not substantially affect protein secretion of T3SS2 (Fig. S3). We also evaluated the effects of pH shifts and Congo red, but they are not influential factors for T3SS2 secretion switch (data are not shown). The results indicate that T3SS2 and T3SS1 have different host cell contact-sensing systems for the regulation of the secretion hierarchy.

10.1128/mBio.01366-18.4 FIG S3  T3SS2 is unresponsive to CaCl_2_ conditions that are known to shift the *Yersinia* T3SS secretion. Download FIG S3, TIF file, 0.4 MB. Copyright © 2018 Tandhavanant et al. 2018 Tandhavanant et al. This content is distributed under the terms of the Creative Commons Attribution 4.0 International license.

**FIG 4:**
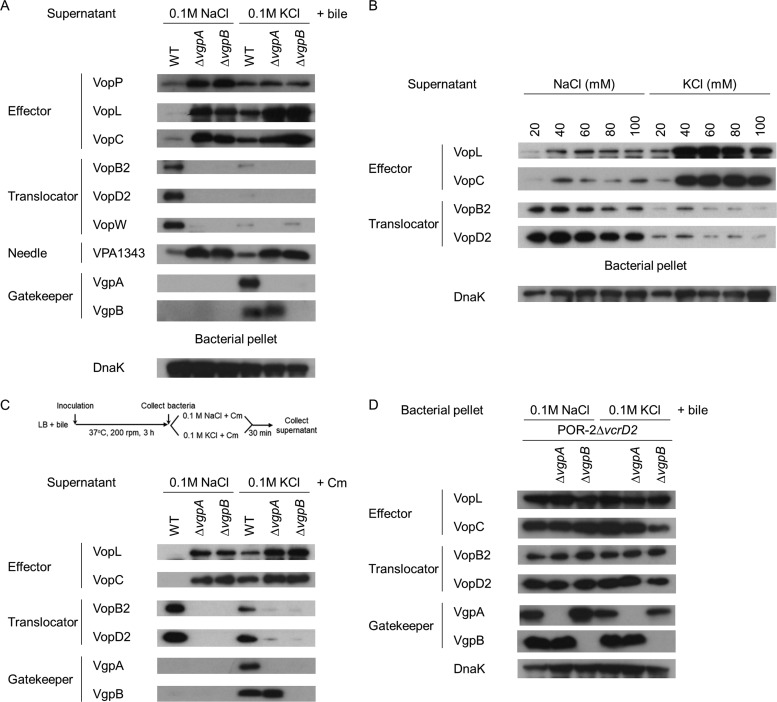
K^+^ triggers VgpA and VgpB secretion and shifts the T3SS2 secretion switch from middle- to late-substrate secretion. (A) KCl-induced promotion of effector secretion and blockage of translocator secretion. The secreted proteins from V. parahaemolyticus strains incubated in LB broth containing 0.1 M concentration of either NaCl or KCl were analyzed by immunoblotting against T3SS2-related proteins. (B) Supernatants from cultures with various concentrations of NaCl or KCl were collected. Proteins from supernatants were precipitated by the addition of trichloroacetic acid (TCA) and analyzed by immunoblotting. (C) New protein synthesis is not required for KCl-induced secretion switching of T3SS2. After 3 h of incubation in the presence of 0.04% bile, bacteria were harvested and resuspended in fresh 0.1 M NaCl or KCl LB broth containing chloramphenicol (Cm). After 30 min of incubation, the supernatants were analyzed by immunoblotting. (D) KCl does not influence the production of T3SS2-related proteins. Isogenic mutant strains of V. parahaemolyticus derived from the T3SS1- and T3SS2-deficient strain (POR-2Δ*vcrD2*) were incubated in LB broth with 0.04% crude bile and 0.1 M NaCl or KCl for 6 h. Bacterial pellets were analyzed by immunoblotting.

Interestingly, we established that the concentration of K^+ ^ions is a potent switching factor of T3SS2. Na^+^ and K^+^ ions are important for homeostasis in eukaryotic cells, and the concentrations of both these cations differ considerably inside and outside cells. The intracellular concentration of K^+^ is 130 to 145 mM, whereas the extracellular value is 3.5 to 5.2 mM (35). Conversely, the concentration of Na^+^ is high (135 to 146 mM) outside the cell but low (25 to 35 mM) inside. This unequal ion distribution is created by the action of the Na^+^−K^+^ pump, which transports three Na^+^ ions out of the cell and two K^+^ ions in. Surprisingly, the levels of effectors secreted by the WT strain increased when the bacteria were cultured in Luria-Bertani (LB) broth supplemented with 0.1 M KCl (K^+^ conditions) instead of 0.1 M NaCl (Na^+^ conditions) (Fig. 4A). On the other hand, the levels of secreted translocators from WT cultured under K^+^ conditions were lower than from bacteria cultured under Na^+^ conditions. This secretion profile of WT bacteria cultured under K^+^ conditions was similar to those of Δ*vgpA* and Δ*vgpB* mutants cultured under Na^+^ conditions. The minimum concentration of K^+ ^ions that was required to shift T3SS2 secretion was 20 mM, which is a far higher concentration than that present in the extracellular milieu (Fig. 4B). The secreted protein profile of the WT also shifted within 30 min after exposure to 0.1 M KCl, even in the presence of the protein synthesis inhibitor chloramphenicol (Fig. 4C).

To clarify whether the shift in secreted protein profile that is observed under Na^+^ and K^+^ conditions contributes to the difference in the levels of expression of T3SS2-related proteins, we determined the levels of T3SS2-related protein production in bacterial pellets from derivatives of strain POR-2Δ*vcrD2*. The production of T3SS2-related proteins—including the effectors (VopL and VopC), translocators (VopB2 and VopD2), and gatekeepers (VgpA and VgpB)—under K^+^ conditions was also comparable to that under Na^+^ conditions (Fig. 4D). Overall, these results indicate that high concentrations of K^+^ alter the secreted protein profile of the WT to that of the Δ*vgpA* or Δ*vgpB* mutant without influencing the levels of production of VgpA or VgpB.

Figure 3A to C show that the secretion of VgpA through T3SS2 occurs when the host cell is in contact with the bacteria. The concentration of K^+ ^ions is low outside the host cell and high inside. Several gatekeepers are secreted in response to stimulation (7, 8). We therefore hypothesized that high concentrations of K^+^ may affect the localization of VgpA and VgpB. Immunoblotting of fractionated bacterial pellets revealed the presence of VgpA in bacterial cytoplasmic and membrane fractions, whereas VgpB was found only in the bacterial cytoplasmic fraction when the bacteria were cultured under Na^+^ conditions (Fig. S4); this localization of VgpA and VgpB was not affected by K^+ ^ions, indicating that exposure to K^+^ did not considerably impact the localization of VgpA or VgpB in bacteria. However, both VgpA and VgpB were detected in the culture supernatant of WT cells cultured under K^+^ conditions (Fig. 4A). Unlike the case of T3SS2 effectors, *vgpB* was needed for VgpA secretion, whereas *vgpA* was not necessary for VgpB secretion. The K^+^-dependent secretion of neither VgpA nor VgpB was affected by the addition of chloramphenicol (Fig. 4C). These findings collectively suggest that high concentrations of K^+^ prompt the T3SS2 secretion switch from the middle substrates to the late substrates.

10.1128/mBio.01366-18.5 FIG S4  K^+^ ions do not considerably impact the localization of VgpA or VgpB in bacterial cells. Download FIG S4, TIF file, 0.3 MB. Copyright © 2018 Tandhavanant et al. 2018 Tandhavanant et al. This content is distributed under the terms of the Creative Commons Attribution 4.0 International license.

### High concentrations of K^+^ in host cells are important for effective translocation of T3SS2 effectors.

**FIG 5:**
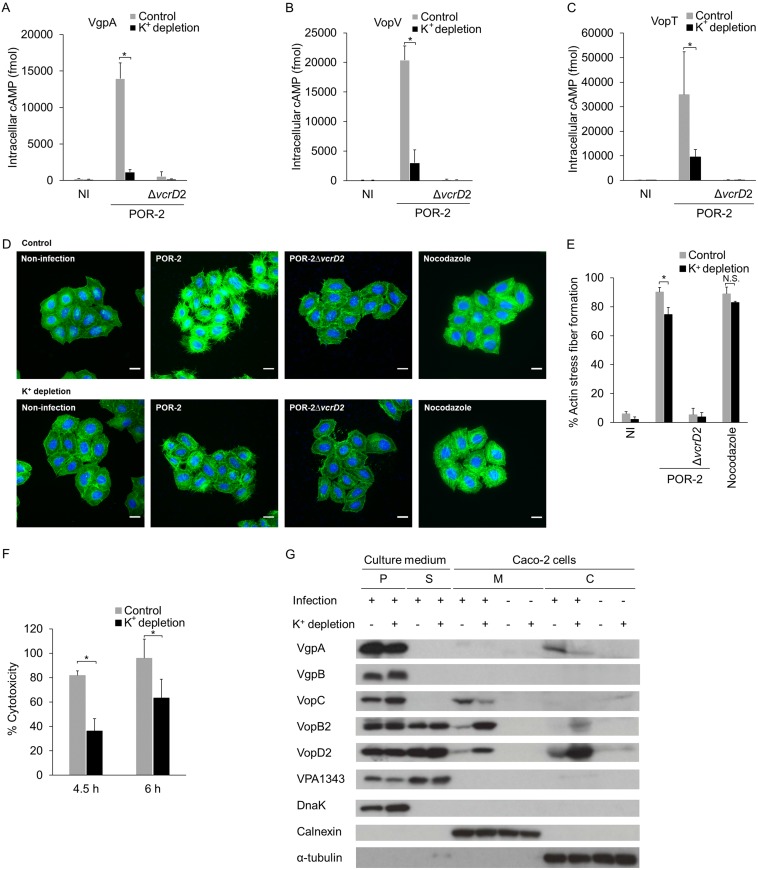
Intracellular K^+^ is important for effective translocation of T3SS2 effectors. (A) K^+^ depletion in host cells significantly decreases the effectiveness of VgpA translocation. Translocation of VgpA into intracellular K^+^-depleted Caco-2 cells was monitored by using a CyaA-based method. Intracellular cAMP levels reflect the translocation of VgpA-CyaA after 1.5 h of infection. The bars are the means from three independent experiments. Error bars indicate SDs. *, *P* ≤ 0.05. (B and C) Translocation of T3SS2 effector (VopV and VopT) into intracellular K^+^-depleted Caco-2 cells. The bars are the means from three independent experiments. Error bars indicate SDs. *, *P* ≤ 0.05 (D) Intracellular K^+^ depletion affects T3SS2-dependent actin stress fiber formation but does not influence nocodazole-induced actin fiber formation. The infected cells were stained with Alexa Fluor 488-phalloidin (F-actin [green]) and Hoechst 33258 (cellular and bacterial DNA [blue]). Bars, 100 µm. (E) Percentage of cells positive for T3SS2-dependent actin stress fiber formation in intracellular K^+^-depleted HeLa cells. The bars are the means from three independent experiments. Error bars indicate SDs. *, *P* ≤ 0.05; N.S., not significant. (F) Effect of K^+^ depletion on T3SS2-dependent cytotoxicity against Caco-2 cells. The bars are the means from three independent experiments. Error bars indicate SDs. *, *P* ≤ 0.05. (G) K^+^ depletion in host cells influences the efficiency of T3SS2-secreted protein translocation. Translocation of T3SS2-related proteins into intracellular K^+^-depleted Caco-2 cells was examined by immunoblotting analysis against fractionated samples (bacterial pellet [P], culture supernatant [S], host membrane [M], and host cytosol [C]). DnaK, calnexin, and tubulin were used as markers of bacteria, host membranes, and the host cytosol, respectively.

To clarify the importance of intracellular K^+^ for effector translocation, we examined the effects of intracellular K^+^ depletion on the translocation of T3SS2 effectors and on T3SS2-dependent biological activities. A K^+^-specific ionophore, valinomycin, and a Na^+^/K^+^-ATPase inhibitor, ouabain, were used to deplete intracellular K^+^. The depletion of intracellular K^+^ did not significantly influence the translocation of T3SS1 effectors, including VP1680 and VPA0450 (Fig. S5A and B). In contrast, the translocations of VgpA (Fig. 5A) and T3SS2 effectors, including VopV and VopT (Fig. 5B and C), decreased significantly. There were no significant differences in cytotoxicity between intracellular K^+^-depleted cells and untreated cells under the infection conditions (Table S2), indicating that cytotoxicity does not affect effector translocation. These results therefore indicate that the concentration of intracellular K^+^ affects the translocation efficiency specific to T3SS2 substrates. We next examined the effect of intracellular K^+^ depletion on T3SS2-dependent biological activities, including T3SS2-dependent stress fiber formation and cytotoxicity. Whereas depletion of intracellular K^+^ did not significantly affect nocodazole-induced actin stress fiber formation, T3SS2-induced stress fiber decreased significantly (Fig. 5D and E). T3SS2-dependent cytotoxicity against Caco-2 cells was significantly delayed in the cells depleted of intracellular K^+^ (Fig. 5F). These results indicate that intracellular concentrations of K^+^ are important for effective translocation of T3SS2 effectors. The reduction of VopC and VgpA translocations into intracellular K^+^-depleted cells was also observed by immunoblotting against fractionated infected cells (Fig. 5G). Although K^+^ depletion did not affect the production of either VopC or VgpA in the suspended bacteria (pellet from culture medium), the translocation of VgpA into the host cytosol and VopC into the host cell membrane decreased in the intracellular K^+^-depleted cells. In contrast to VopC and VgpA, the translocation of the translocators (VopB2 and VopD2) into the host cell membrane and cytosol was promoted by K^+^ depletion. The inverse relationship between the translocated quantities of effectors and translocators caused by the depleted intracellular concentration of K^+^ correlated well with the results that were observed under *in vitro* growing conditions, including K^+^ (Fig. 4A). These results strongly indicate that intracellular concentrations of K^+^ indeed affect the efficiency of effector translocation by switching T3SS2 substrate secretion.

10.1128/mBio.01366-18.6 FIG S5  Depletion of intracellular K^+^ did not significantly influence the translocation of T3SS1 effectors, including VP1680 (A) and VPA0450 (B). Download FIG S5, TIF file, 0.3 MB. Copyright © 2018 Tandhavanant et al. 2018 Tandhavanant et al. This content is distributed under the terms of the Creative Commons Attribution 4.0 International license.

10.1128/mBio.01366-18.8 TABLE S2  Cytotoxicity against Caco-2 cells by V. parahaemolyticus (POR-2) under K^+^ depletion condition after 1.5-h infection. Download TABLE S2, DOCX file, 0.01 MB. Copyright © 2018 Tandhavanant et al. 2018 Tandhavanant et al. This content is distributed under the terms of the Creative Commons Attribution 4.0 International license.

## DISCUSSION

Several *in vivo* studies have revealed that T3SS2 is the main contributor to the enteropathogenicity of V. parahaemolyticus (18, 19). T3SS2-related genes, such as structural components, transcriptional regulators, translocators, and effectors, have been characterized (21, 23, 36–39). However, numerous genes with unidentified functions are also found in this region (16). In addition, the protein components of the T3SS2 that have a role in substrate switching, such as molecular ruler (which regulates the length of the needle complex of the T3SS) and gatekeeper (which switches translocator and effector secretions upon contact with host cells), have not been identified. The precise mechanism of action of T3SS2 therefore remains unknown. In this study, we have identified and characterized two hypothetical genes encoded on Vp-PAI, *vpa1360* (VgpA) and *vpa1359* (VgpB), as gatekeeper proteins of T3SS2.

Gatekeeper blocks effector secretion until the host cell is contacted, but its role in translocator secretion is distinct in each bacterium (40). Most gatekeeper mutants (*invE*, *sepL*, and *ssaL* mutants) cannot secrete translocators (6, 11, 30), whereas gatekeeper mutants of *Yersinia* spp. demonstrate increased translocator secretion (41, 42). However, results from gatekeeper mutants of *Shigella* were ambiguous. In an *mxiC* mutant, codons 19 to 335 of *mxiC* were replaced by a *ble* cassette and resulted in no defects in translocator secretion (7). In contrast, a nonpolar *mxiC* knockout mutant exhibits impaired translocator secretion when induced with Congo red (32). Our study demonstrates that VgpA- and VgpB-defective strains secrete effectors extremely well, whereas translocator secretion is abolished when strains are cultured in LB broth. Conversely, gatekeeper mutants secrete translocators (VopD2 and VopW) in DMEM. However, the gatekeeper mutants unable to form a complete translocator pore for delivery of effectors lack VopB2 secretion. The ambiguous secretion of translocators (VopD2 and VopW), which similar to *mxiC* mutants of *Shigella*, need further investigation of the precise mechanism of gatekeepers and the interaction between gatekeepers and other T3SS2 proteins. Besides, the alteration of T3SS2 secretion by gatekeeper mutants is independent of bacterial growth and membrane damage. It is thus implied that T3SS2 gatekeepers are necessary for translocator (VopB2) secretion and for suppression of effector secretion.

Gatekeeper protein is thought to be located at the cytoplasmic base of the injectisome, and the proteins are released upon host cell contact (7, 8). Several T3SSs of other bacteria sense the concentrations of cations in the intracellular environment to recognize attachment to the host cell. The T3SSs of *Yersinia* spp. and enteropathogenic *Escherichia coli* (EPEC) sense low intracellular concentrations of Ca^2+^ (31, 43, 44), whereas the *Salmonella* SPI2 T3SS distinguishes the pH of the cytoplasm from that of the phagolysosome (11). The artificial stimulator (Congo red), similar to Ca^2+^ and H^+^, induces *Shigella* effector secretion (33). In our investigation, T3SS2 is insensitive to low concentrations of Ca^2+^, implying the existence of a unique T3SS activation system. We have determined that K^+^ ions activate effector secretion without host cell contact and new protein synthesis, similar to the behavior observed in gatekeeper mutants. In addition, K^+^ also induces VgpA and VgpB secretion and facilitates the translocation of VgpA, similar to the cases of YopN, MxiC, and SsaL, which are secreted or released from complexes upon exposure to specific stimulators (8, 11, 40). Although VgpB is secreted in response to K^+^ ions and translocated into host cells, the VgpB translocation is delayed and the level of translocation is low compared with VgpA by CyaA-based translocation assay. Therefore, we could not detect VgpB in the fractions of infected host cells. These results indicate that K^+^ either inactivates VgpA and VgpB or releases these proteins from the injectisome for the activation of effector secretion.

The sensing of intracellular K^+^ concentration as a signal for host cell contact seems reasonable because the concentrations of K^+^ inside and outside the cell differ considerably. Indeed, we revealed that intracellular K^+^, a host factor, is critical for converting T3SS substrate secretion from middle substrates (translocators) to late substrates (effectors). In addition, this signal transduction facilitates the effective translocation of T3SS2 effectors into host cells. Although details of the molecular mechanisms of intracellular K^+^ sensing by T3SS2 and substrate switching via VgpA and VgpB remain to be elucidated, further investigations should provide new insight into how pathogens recognize attachment to the cell of the host to exert pathogenesis.

## MATERIALS AND METHODS

### Bacterial strains and culture conditions.

Vibrio parahaemolyticus RIMD2210633 (Kanagawa phenomenon [KP] positive, serotype O3:K6) was obtained from the Pathogenic Microbes Repository Unit, International Research Center for Infectious Diseases, Research Institute for Microbial Diseases, Osaka University, Osaka, Japan. Escherichia coli DH5α and SM10λ*pir* were used for DNA manipulation. Strains and plasmids are listed in Table S3 in the supplemental material. The V. parahaemolyticus strains were grown in Luria-Bertani (LB) broth containing 0.5% NaCl, 0.1 M NaCl, or 0.1 M KCl at 37°C. A four-primer PCR technique was used to engineer an in-frame deletion mutant of *vgpA* and *vgpB* as described previously (19). The primers are listed in Table S4.

10.1128/mBio.01366-18.9 TABLE S3  Bacterial strains and plasmids used in this study. Download TABLE S3, DOCX file, 0.03 MB. Copyright © 2018 Tandhavanant et al. 2018 Tandhavanant et al. This content is distributed under the terms of the Creative Commons Attribution 4.0 International license.

10.1128/mBio.01366-18.10 TABLE S4  Sequences of the primers used for gene deletion. Download TABLE S4, DOCX file, 0.01 MB. Copyright © 2018 Tandhavanant et al. 2018 Tandhavanant et al. This content is distributed under the terms of the Creative Commons Attribution 4.0 International license.

### Bacterial growth curves.

An overnight culture (40 µl) was inoculated in fresh medium (4 ml) and then incubated at 37°C. The bacterial growth was monitored every 30 min for 7.5 h by measuring the optical density at 600 nm. Each data point is given as the average optical density from three independent experiments. The error bars depict standard deviations (SDs).

### Assessment of live-dead bacteria.

An overnight culture (40 µl) was inoculated in fresh medium (4 ml) and then incubated at 37°C for 6 h. Bacteria were collected from a sample of the culture (100 µl) and washed with 0.85% NaCl. The bacteria were stained with SYTO9 (green fluorescence) for all bacteria and propidium iodide (red fluorescence) for bacteria with damaged membrane, by using a LIVE/DEAD BacLight bacterial viability kit (Molecular Probes), and then incubated at room temperature in the dark for 15 min. The emission fluorescence intensity at 528 ± 20 nm (green fluorescence) and 590 ± 35 nm (red fluorescence) with excitation at 485 ± 20 nm of the mixtures were measured. The percentage of live mutants was calculated and compared with the parental strain. The heat-killed bacteria were used as 100% dead bacteria. Live and dead bacteria of the parental strain were mixed to achieve various proportions for fluorescence measurement. The ratio of green to red fluorescence emission is proportional to the relative number of the live parental strain that was used for calculating the percentage of live derivative strains. The average ratios from three independent experiments are plotted on the bar graphs. The error bars depict SDs.

### Cytotoxicity.

Caco-2 cells were maintained in Dulbecco’s modified Eagle’s medium (DMEM) supplemented with 10% fetal bovine serum and 100 µg/ml gentamicin at 37°C with 5% CO_2_. Caco-2 cells (1.5 × 10^4^ cells) were seeded into each well of a 96-well plate and incubated for 2 days. Cells were washed twice with phenol red-free DMEM and infected with V. parahaemolyticus at a multiplicity of infection (MOI) of 100 for 1.5, 3, 4.5, or 6 h.

Cytotoxicity was evaluated by measuring lactate dehydrogenase (LDH) release into the culture supernatant with a CytoTox96 nonradioactive cytotoxicity assay (Promega). The test was performed according to the manufacturer’s instructions.

### Enterotoxicity.

Rabbit ileal loop tests were performed as previously described (37). Bacterial cells (10^9^ CFU) were collected and suspended in 1 ml LB broth. The bacterial suspension was injected into the ligated ileal loop of rabbits, and the amount of fluid in each loop was measured 15 h after infection. The fluid accumulation (FA) ratio was calculated as the volume of fluid (in milliliters) per length of ligated rabbit small intestine (in centimeters). All animal experiments were performed according to an experimental protocol approved by the Ethics Review Committee for Animal Experimentation of the Research Institute for Microbial Diseases (Osaka University, Japan).

### CyaA-based translocation assay.

Caco-2 cells were infected with V. parahaemolyticus harboring plasmids expressing each protein C-terminally fused to the catalytic domain of adenylate cyclase toxin (CyaA) (37) at an MOI of 100 for 1.5 h or 3 h. The translocated CyaA-fused proteins increased the levels of intracellular cAMP by cyclizing AMP. The levels of intracellular cAMP in infected cells were determined using a cAMP Biotrak enzyme immunoassay system (GE Healthcare), according to the manufacturer’s instructions.

### Analysis of secreted proteins.

V. parahaemolyticus strains were grown overnight in LB broth containing 0.5% NaCl. The overnight cultures were diluted into DMEM or LB with 0.5% NaCl, 0.1 M NaCl, or 0.1 M KCl and then incubated for 6 h. To examine the effect of protein synthesis inhibition on protein secretion, overnight cultures were diluted into LB broth supplemented with 0.04% crude bile and then incubated at 37°C with shaking at 200 rpm for 3 h. Bacteria were collected from 6-ml cultures and then resuspended with 1 ml of fresh medium containing 12.5 µg/ml chloramphenicol (Cm). The bacteria were further incubated at 37°C for 30 min. Culture supernatants were collected by centrifugation at 1,580 × *g* for 10 min and then filtered through a 0.2-µm membrane. Proteins in the supernatant were precipitated with trichloroacetic acid (TCA) at a final concentration of 14% (vol/vol) on ice for 1 h, followed by centrifugation at 15,800 × *g* and 4°C for 30 min. The pellets were washed with cold 100% acetone and then dried at room temperature for 15 min. Protein pellets were suspended in Laemmli buffer and then heated at 95°C for 5 min. The samples were separated and analyzed by SDS-PAGE and then silver stained or analyzed by immunoblotting.

### Immunoblot analysis.

Samples for immunoblot analysis were separated by SDS-PAGE. After electrotransfer, the membranes were probed with the sera of rabbits immunized with these proteins (e.g., VopP, VopL, VopC, VopB2, VopD2, VopW, VPA1343, VgpA, VgpB, maltose-binding protein (MBP or MalE), DnaK, or OmpA), or a mouse monoclonal antibody against calnexin (Chemicon), or alpha-tubulin (Sigma-Aldrich). The samples were then probed with horseradish peroxidase-conjugated goat anti-rabbit antibody (Zymed) or horseradish peroxidase-conjugated rabbit anti-mouse antibody (Zymed), respectively. The blots were developed with an ECL Western blotting kit (GE Healthcare).

### Bacterial fractionation.

V. parahaemolyticus was grown in LB broth at 37°C with shaking at 200 rpm for 5 h. Bacterial pellets were collected from 500 µl of culture and mixed with 200 µl of Laemmli buffer to make whole-cell samples. Fractionation was performed using 2 ml of culture. Bacterial pellets were suspended in 200 µl of periplasting buffer (200 mM Tris-HCl [pH 7.5], 20% sucrose, and 1 mM EDTA). Bacterial suspensions were incubated at room temperature for 5 min, and 200 µl of ice-cold distilled water was then added. This mixture was incubated on ice for 5 min. Periplasmic fractions were collected by centrifugation at 13,400 × *g* for 2 min and then mixed with an equal volume of 2× Laemmli buffer. The pellets were washed with 1 ml of periplasting buffer. Periplastic bacteria were lysed with 400 µl of Benzonase solution (50 mM Tris-HCl [pH 7.5], 2 mM MgCl_2_, 500 U/ml Benzonase, and protease inhibitor cocktail) and then sonicated for 5 min. Intact bacteria were removed by centrifuging the samples twice at 13,400 × *g* for 2 min. Cytoplasmic fractions were separated by ultracentrifugation at 98,560 × *g* and 4°C for 1 h and then mixed with an equal volume of 2× Laemmli buffer. The pellet was washed with 800 µl of 50 mM Tris-HCl (pH 7.5) containing protease inhibitor cocktail and ultracentrifuged at 98,560 × *g* and 4°C for 1 h. The pellet was collected and suspended in 80 µl of Laemmli buffer to produce a 10× membrane fraction.

### Fractionation of infected host cells.

Fractionation of infected cells was performed as previously described (37). Briefly, V. parahaemolyticus-infected Caco-2 cells were washed with ice-cold phosphate-buffered saline (PBS). Homogenization buffer (3 mM imidazole, 250 mM sucrose, and 0.5 mM EDTA [pH 8.0]) was added, and cells were then lysed by passage through a 22-gauge needle. Intact cells and bacteria were removed by centrifugation at 880 × *g* and 4°C for 15 min. Supernatants were collected for separation into membrane (pellet) and cytoplasmic (supernatant) fractions by ultracentrifugation at 75,460 × *g* and 4°C for 20 min. Fractions were analyzed by immunoblotting as described above.

### K^+^ depletion of the host cells.

Caco-2 or HeLa cells were seeded into tissue culture plates and maintained at 37°C and 5% CO_2_ for 2 days. The cells were washed twice and maintained with phenol red-free DMEM. The cells were treated with 10 µM valinomycin (Abcam) and 10 µM ouabain (Sigma-Aldrich) in DMEM for 20 min. The cells were then washed and maintained with phenol red-free DMEM until use in experiments.

### Immunofluorescence assay.

HeLa cells were infected with V. parahaemolyticus strains at an MOI of 100 for 3 h. After infection, the cells were washed with PBS and then fixed with 4% paraformaldehyde in PBS. The fixed cells were probed with Alexa Fluor 488-phalloidin (Invitrogen) to stain F-actin and Hoechst 33258 (Sigma-Aldrich) to highlight host cell and bacterial DNA. Images were captured with a fluorescence microscope (Nikon).

### Statistical analysis.

All bar graphs and error bars show the means and SDs from three independent experiments, except enterotoxicity for which two independent experiments were performed. A Student’s *t* test was used for statistical analysis. *P* values of ≤0.05 were considered to be statistically significant.

10.1128/mBio.01366-18.1 TEXT S1  Detailed supplemental material legends and references. Download TEXT S1, DOCX file, 0.02 MB. Copyright © 2018 Tandhavanant et al. 2018 Tandhavanant et al. This content is distributed under the terms of the Creative Commons Attribution 4.0 International license.
