# Supplementary figures and images for: Vibrio parahaemolyticus Senses Intracellular K+ To Translocate Type III Secretion System 2 Effectors Effectively
Source: mBio. 2018 Jul 24;9(4):e01366-18. doi: 10.1128/mBio.01366-18 (PMC6058294; doi:10.1128/mBio.01366-18)

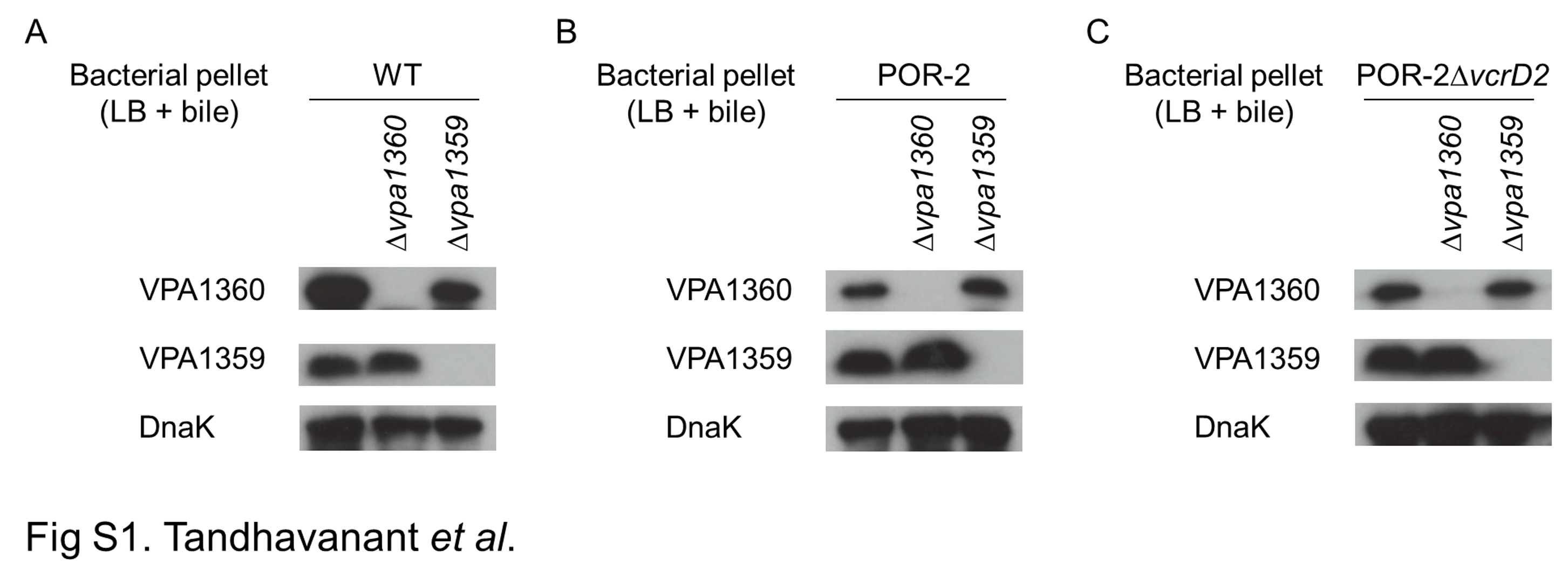

Supplement: FIG S1 [file mbo004184001sf1.tif]

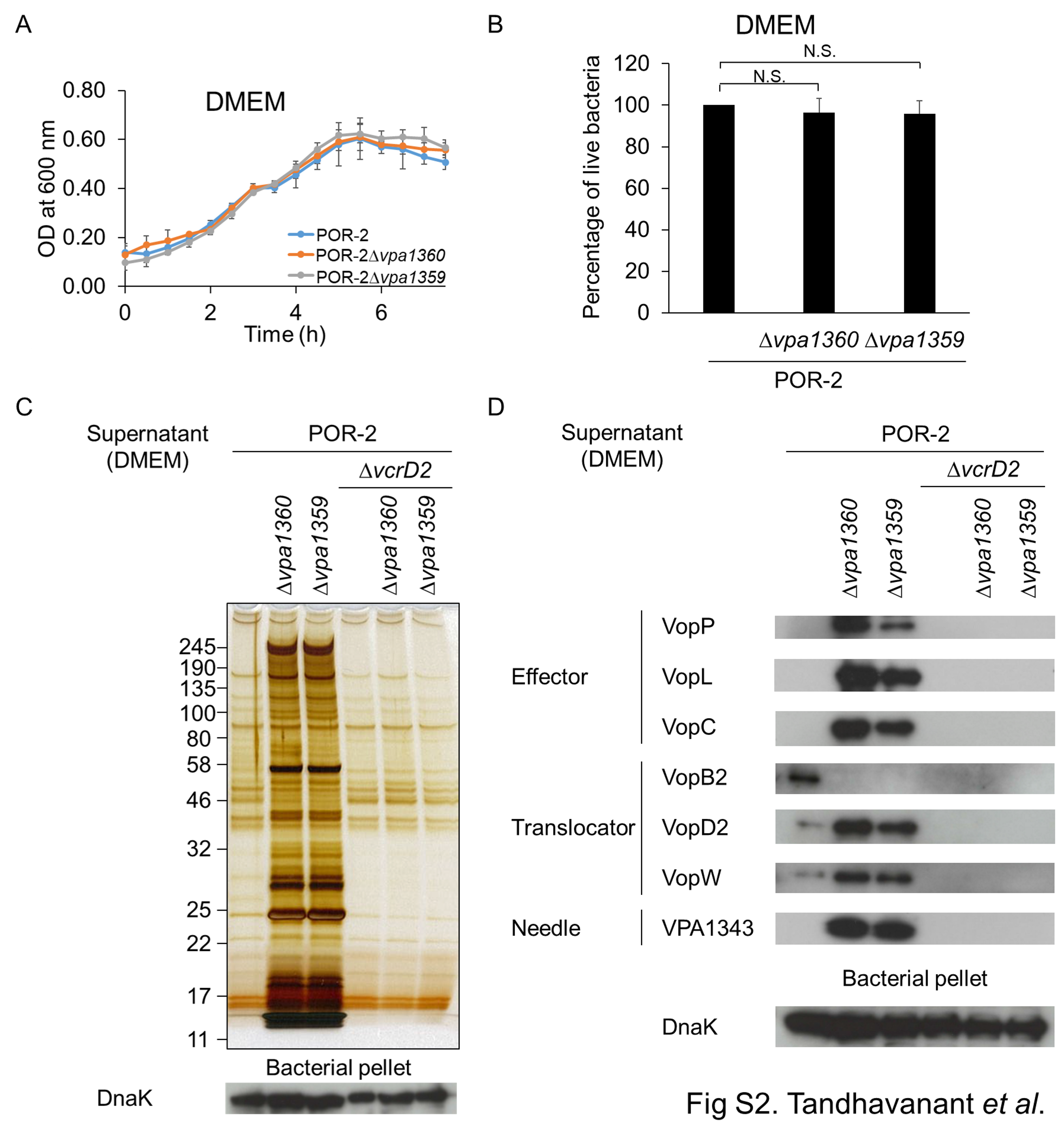

Supplement: FIG S2 [file mbo004184001sf2.tif]

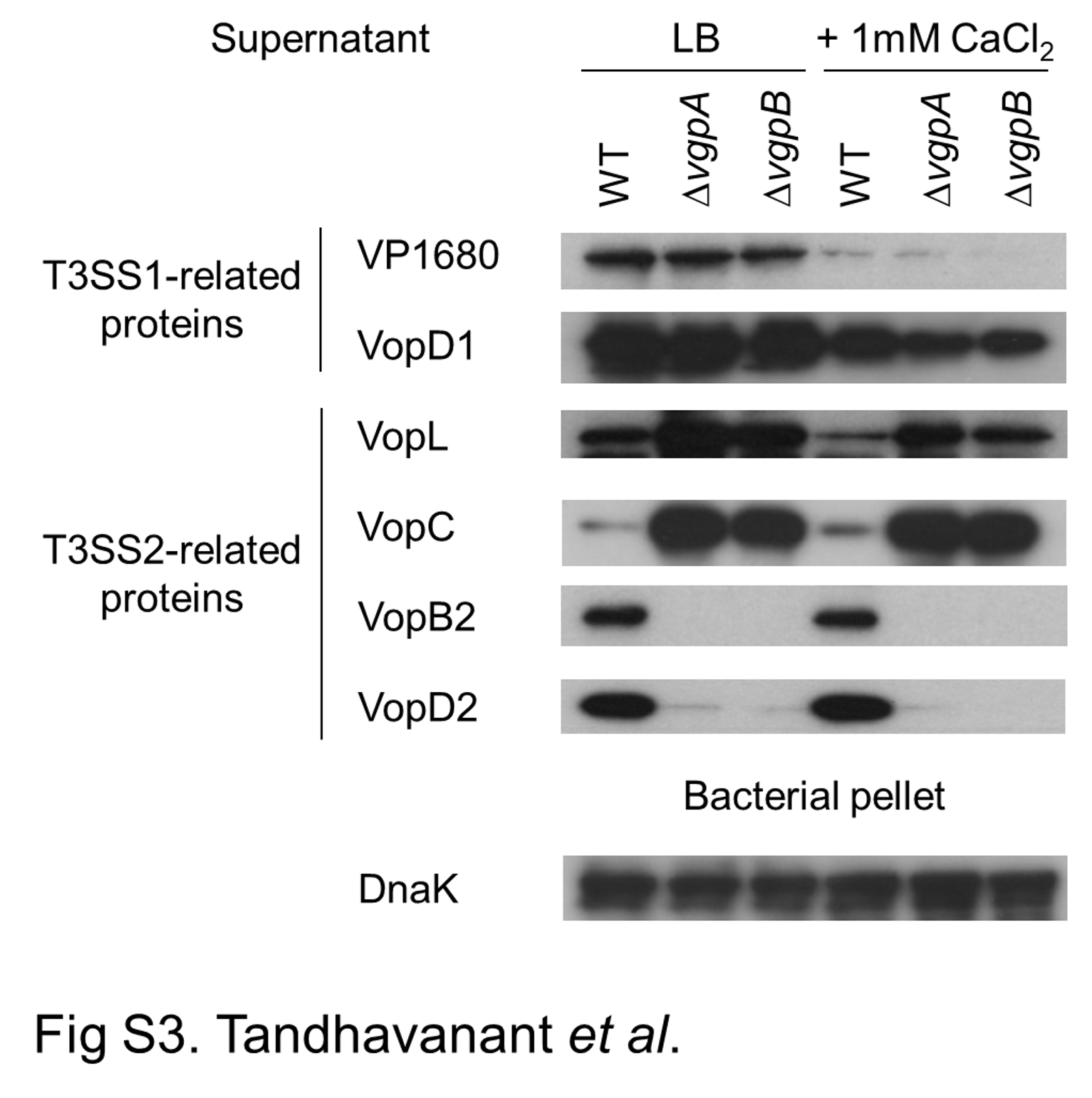

Supplement: FIG S3 [file mbo004184001sf3.tif]

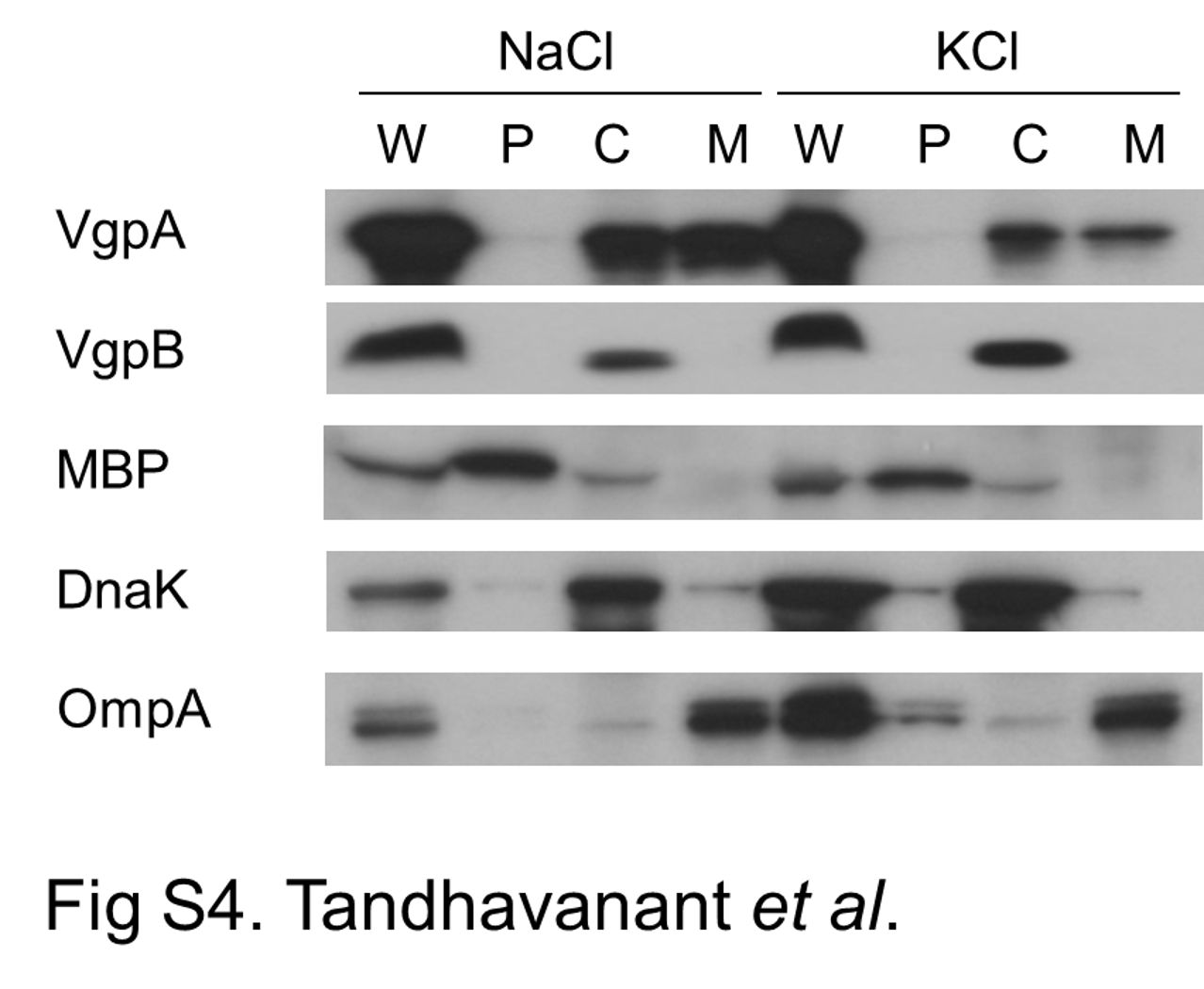

Supplement: FIG S4 [file mbo004184001sf4.tif]

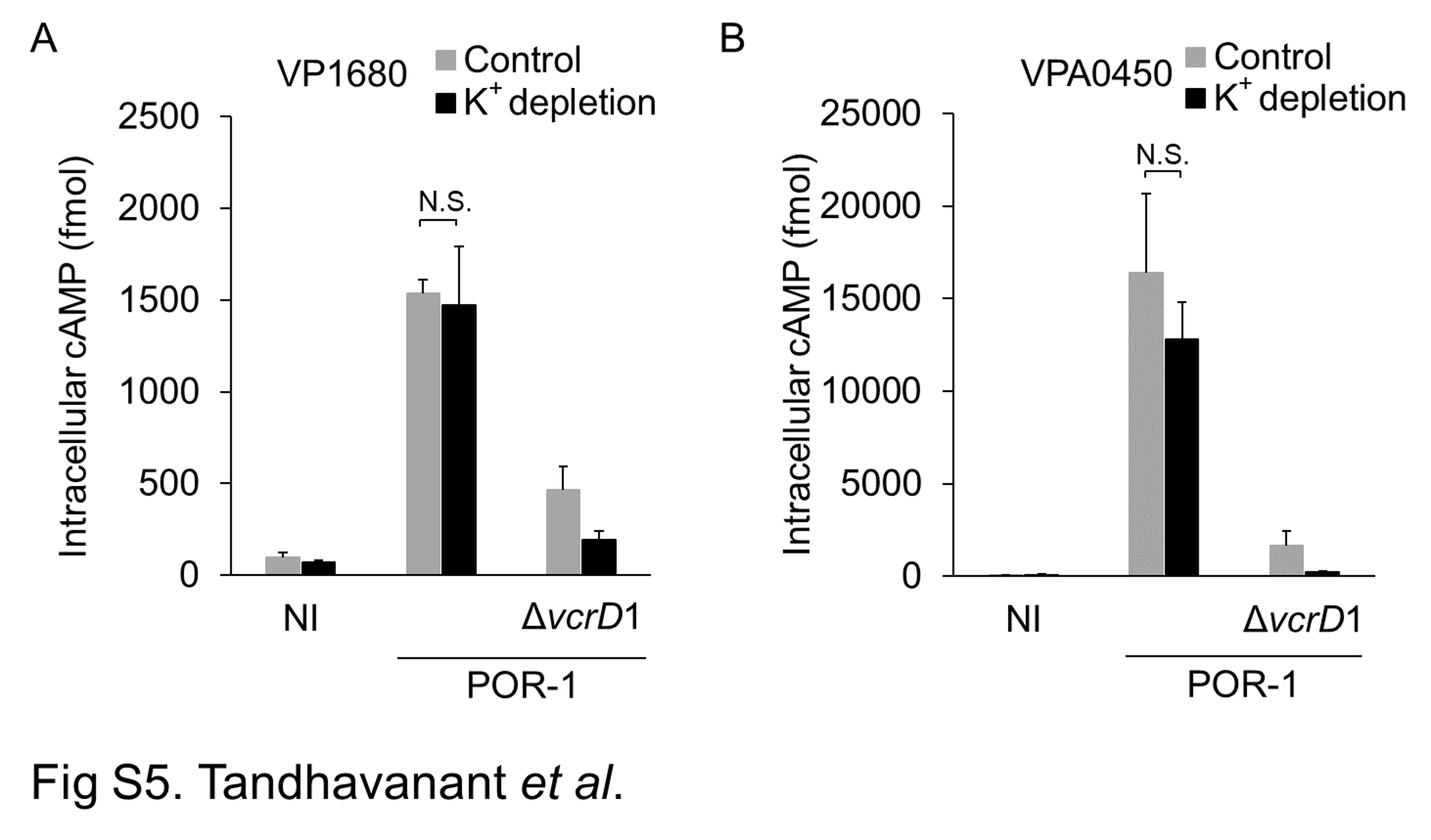

Supplement: FIG S5 [file mbo004184001sf5.tif]
