# Supplementary material for: Vibrio parahaemolyticus Senses Intracellular K+ To Translocate Type III Secretion System 2 Effectors Effectively
Source: mBio. 2018 Jul 24;9(4):e01366-18. doi: 10.1128/mBio.01366-18 (PMC6058294; doi:10.1128/mBio.01366-18)
Supplement: TABLE S1 [file mbo004184001st1.docx]

**Table S1. Cytotoxicity against Caco-2 cells by *V. parahaemolyticus* (POR-2) and derivative strain after infection of 1.5 or 3 h**

| % Cytotoxicity (mean ± SD) |  |  |
| --- | --- | --- |
| Strain | Time of infection (h) | |
|  | 1.5 h | 3 h |
| POR-2 | 0.12 ± 0.16 | 1.60 ± 2.7 |
| POR-2∆*vcrD2* | 0.00 | 0.15 ± 0.30 |
| POR-2∆*vpa1360* | 0.00 | 1.83 ± 3.65 |
| POR-2∆*vpa1359* | 1.15 ± 1.24 | 1.20 ± 1.61 |
| N.S. for all comparisons with POR-2 by Student's t-test | | |
