# Supplementary material for: Vibrio parahaemolyticus Senses Intracellular K+ To Translocate Type III Secretion System 2 Effectors Effectively
Source: mBio. 2018 Jul 24;9(4):e01366-18. doi: 10.1128/mBio.01366-18 (PMC6058294; doi:10.1128/mBio.01366-18)
Supplement: TABLE S2 [file mbo004184001st2.docx]

**Table S2. Cytotoxicity against Caco-2 cells by *V. parahaemolyticus* (POR-2) under K^+^ depletion condition after infection of 1.5 h**

| Condition | % Cytotoxicity (mean ± SD) |  |
| --- | --- | --- |
| Control | 1.20 ± 1.15 |  |
| K^+^ depletion | 2.02 ± 1.81 |  |
| N.S. for comparisons between 2 conditions by Student's t-test | | |
