# Supplementary material for: Vibrio parahaemolyticus Senses Intracellular K+ To Translocate Type III Secretion System 2 Effectors Effectively
Source: mBio. 2018 Jul 24;9(4):e01366-18. doi: 10.1128/mBio.01366-18 (PMC6058294; doi:10.1128/mBio.01366-18)
Supplement: TABLE S3 [file mbo004184001st3.docx]

**Table S3. Bacterial strains and plasmids**

| **Strain or Plasmid** | | **Description** | **Source or references** |
| --- | --- | --- | --- |
| ***Vibrio parahaemolyticus*** | |  |  |
|  | RIMD2210633 (wild-type; WT) | Clinical isolate; KP-positive; serotype O3:K6 | (1) |
|  | POR-1 | *tdhAS* null mutant strain: *tdhAS* deletion mutant derived from WT | (2) |
|  | POR-2 | TDH- and T3SS1-deficient strain: *vcrD1* deletion mutant derived from POR-1 strain | (3) |
|  | POR-2∆*vcrD2* | TDH-, T3SS1- and T3SS2-deficient strain: *vcrD2* deletion mutant derived from POR-2 | (4) |
|  | WT∆*vscN2* | T3SS2-deficient strain; *vscN2* deletion mutant derived from WT | (5) |
|  | ∆*vgpA* (*vpa1360*) | VgpA-deficient strain; *vgpA* deletion mutant derivative of WT | This study |
|  | ∆*vgpB* (*vpa1359*) | VgpB-deficient strain; *vgpB* deletion mutant derivative of WT | This study |
|  | POR-2∆*vgpA* (*vpa1360*) | TDH-, T3SS1- and VgpA-deficient strain; *vgpA* deletion mutant derivative of POR-2 | This study |
|  | POR-2∆*vgpB* (*vpa1359*) | TDH-, T3SS1- and VgpB-deficient strain; *vgpB* deletion mutant derivative of POR-2 | This study |
|  | POR-2∆*vcrD2-vgpA* (*vpa1360*) | TDH-, T3SS1-, T3SS2- and VgpA-deficient strain; *vgpA* deletion mutant derivative of POR-2∆*vcrD2* | This study |
|  | POR-2∆*vcrD2-vgpB* (*vpa1359*) | TDH-, T3SS1-, T3SS2- and VgpB-deficient strain; *vgpB* deletion mutant derivative of POR-2∆*vcrD2* | This study |
|  |  |  |  |
| ***Escherichia coli*** | |  |  |
|  | DH5α | F^-^ ɸ80*lacZ*∆M15∆(*lacZYA-argF*)*U169* *deoR recA1 endA1 hsdR17* *phoA supE44 thi-1gyrA96* *relA1* λ*^-^* | Laboratory collection |
|  | SM10 λ*pir* | *thi thr leu tonA lacY supE recA*::RP4-2Tc::Mu λ*pir* R6K | Laboratory collection |
|  |  |  |  |
| **Plasmids** | |  |  |
|  | pCR2.1-TOPO | Multicopy (ColE1 *ori*) TA cloning vector, Amp^r^ | Novagen |
|  | pYAK1 | R6K-*ori* suicide vector containing *sacB* gene for gene replacement, Cm^r^ | (4) |
|  | pYAK1-∆*vgpA* | Derivative of suicide vector pYAK1 for generating the *vgpA* (*vpa1360*) deletion mutant | This study |
|  | pYAK1-∆*vgpB* | Derivative of suicide vector pYAK1 for generating the *vgpB (vpa1359)* deletion mutant | This study |
|  | pSA19CP-MCS | Complement vector for *V. parahaemolyticus*, Cm^r^ | (4) |
|  | pSA-*tdh*P | pSA19CP-MCS containing *tdh* promoter | (4) |
|  | pSA-*vgpA* (*vpa1360*) | pSA-*tdh*P containing full-length *vgpA* gene | This study |
|  | pSA-*vgpB* (*vpa1359*) | pSA-*tdh*P containing full-length *vgpB* gene | This study |
|  | pSA-*cyaA* | pSA-*tdh*P containing *cyaA* gene (4–1216 bp) | (6) |
|  | pSA-*vopV*-*cyaA* | pSA-*cyaA*, *cyaA* C-terminally fused with *vopV* (from 1 to 498 bp) | (7) |
|  | pSA-v*opT*-*cyaA* | pSA-*cyaA*, *cyaA* C-terminally fused with *vopT* (from 1 to 195 bp) | (4) |
|  | pSA-*vpgA*-*cyaA* | pSA-*cyaA*, *cyaA* C-terminally fused with *vgpA* (from 1 to 498 bp) | This study |
|  | pSA-*yopN*P-*cyaA* | pSA-*cyaA* containing *yopN* promoter | This study |
|  | pSA-*yopN*P-*vpa0450*-*cyaA* | pSA-*yopN*P-*cyaA*, *cyaA* C-terminally fused with *vpa0450* (from 1 to 498 bp) | This study |
|  | pSA-*yopN*P-*vp1680*-*cyaA* | pSA-*yopN*P-*cyaA*, *cyaA* C-terminally fused with *vp1680* (from 1 to 498 bp) | This study |
