# Supplementary material for: Vibrio parahaemolyticus Senses Intracellular K+ To Translocate Type III Secretion System 2 Effectors Effectively
Source: mBio. 2018 Jul 24;9(4):e01366-18. doi: 10.1128/mBio.01366-18 (PMC6058294; doi:10.1128/mBio.01366-18)
Supplement: TABLE S4 [file mbo004184001st4.docx]

**Table S4. Sequence of the primers for gene deletion**

| Primers | Sequence (5’-3’) |
| --- | --- |
| *vgpA* gene deletion | |
| *∆vgpA*-1 | CTGCAGCAGTGCTCAATTGCAACAAGGTCGTACTGC |
| *∆vgpA*-2 | AGGGCGGAGCGAGTATCGGATGTTTCTCGCTAAGTGGATGCAGAGTCATTGAACGACGCTCAG |
| *∆vgpA*-3 | CTGAGCGTCGTTCAATGACTCTGCATCCACTTAGCGAGAAACATCCGATACTCGCTCCGCCCT |
| *∆vgpA*-4 | GGATCCAGCGTTGTCTGTTTCAAGTTGAAATGCTTCATC |
| *vgpB* gene deletion | |
| *∆vgpB*-1 | GCATGCGGATGCAGAGTCATTGAACGACGCTCAGTTGTG |
| *∆vgpB*-2 | CGAATCGAATTAGTTGGAACGGCTTCTCCGATAGCCAATAATCCATGAGTTCAATGCAAGTATACCAATTAC |
| *∆vgpB*-3 | GTAATTGGTATACTTGCATTGAACTCATGGATTATTGGCTATCGGAGAAGCCGTTCCAACTAATTCGATTCG |
| *∆vgpB*-4 | AGATCTAGATAAGATAGATATTTCTCATCCATATGAG |
