# Supplementary material for: Vibrio parahaemolyticus Senses Intracellular K+ To Translocate Type III Secretion System 2 Effectors Effectively
Source: mBio. 2018 Jul 24;9(4):e01366-18. doi: 10.1128/mBio.01366-18 (PMC6058294; doi:10.1128/mBio.01366-18)
Supplement: TEXT S1 [file mbo004184001s1.docx]

**Supplemental Information**

**Supplemented figure legends**

**FIG S1** No polar effect from deletion of *vpa1360* and *vpa1359*. Immunoblot analysis of VPA1360 and VPA1359 production from bacteria lysate of mutants derived from *V. parahaemolyticus* RIMD2210633 wild-type (WT; A), POR-2 (WTΔ*tdhAS-vcrD1*; B) and POR-2Δ*vcrD2* (WTΔ*tdhAS-vcrD1-vcrD2*; C)

**FIG S2** *vpa1360* and *vpa1359* mutants alter a pattern of T3SS2 secretion from parental strain without difference of bacterial growth or number of bacteria with damaged membrane. (A) Growth curve of *vpa1360* and *vpa1359* mutants and parental strains cultured in DMEM. The bacteria were cultured at 37°C, and the optical density at 600 nm was measured every 30 min. The data points present mean of three independent experiments. The error bars represent SD. (B) Analysis of live *V. parahaemolyticus* and derivative strains. The bacterial suspension from cultures in DMEM for 6 h was stained with LIVE/DEAD *Bac*Light Bacterial Viability Kit (Molecular Probe). Percentage of live derivative strains was compared with the parental strain. The heat-killed bacteria were used as 100% dead bacteria. The bar graphs present the mean of three independent experiments. Error bars indicate SDs (* *P* ≤ 0.05; N.S., not significant). (C) Silver staining of proteins secreted from *V. parahaemolyticus* cultured in DMEM for 6 h. (D) Effect of *vpa1360* and *vpa1359* gene deletions on secretion of T3SS2 proteins in DMEM. Immunoblotting of T3SS2-secreted proteins (effectors, translocators, and needle) from the supernatant of cultures grown in DMEM for 6 h.

**FIG S3** T3SS2 is unresponsive to CaCl_2_ conditions that are known to shift the *Yersinia* T3SS secretion. CaCl_2_ has no impact on the secretion of T3SS2-related proteins. Culture supernatants from bacterial cultures grown for 6 h in the presence or absence of 1 mM CaCl_2_ were collected, and proteins in the supernatant were then precipitated with TCA. Samples were analyzed by immunoblotting.

**FIG S4** K^+^ ions do not considerably impact the localization of VgpA or VgpB in bacterial cells. Bacterial pellets were fractionated into three fractions, the periplasmic (P), cytoplasmic (C) and membrane (M) fractions. VgpA and VgpB were detected in each bacterial fraction by immunoblotting. MBP, DnaK, and OmpA are markers for the periplasm, cytoplasm and membrane, respectively. The “W” indicates samples from whole-bacterial lysates.

**FIG S5** Depletion of intracellular K^+^ did not significantly influence the translocation of T3SS1 effectors, including VP1680 (A) and VPA0450 (B), respectively. Intracellular K^+^-depleted Caco-2 cells were infected for 1.5 h with POR-1 (WT∆*tdhAS*) harboring pSA-*vp1680*-*cyaA* or pSA-*vpa0450*-*cyaA*. The efficiency of translocation of CyaA-fused effectors into the host cytosol was evaluated by measuring the intracellular level of cAMP. The graph presents the mean ± SD of three independent experiments (N.S., not significant).

**Supplemental references**

1. Makino K, Oshima K, Kurokawa K, Yokoyama K, Uda T, Tagomori K, Iijima Y, Najima M, Nakano M, Yamashita A, Kubota Y, Kimura S, Yasunaga T, Honda T, Shinagawa H, Hattori M, Iida T. 2003. Genome sequence of *Vibrio* *parahaemolyticus*: A pathogenic mechanism distinct from that of *V*. *cholerae*. Lancet 361:743-749.

2. Park KS, Ono T, Rokuda M, Jang MH, Iida T, Honda T. 2004. Cytotoxicity and enterotoxicity of the thermostable direct hemolysin-deletion mutants of *Vibrio* *parahaemolyticus*. Microbiol Immunol 48:313-318.

3. Park KS, Ono T, Rokuda M, Jang MH, Okada K, Iida T, Honda T. 2004. Functional characterization of two type III secretion systems of *Vibrio* *parahaemolyticus*. Infect Immun 72:6659-6665.

4. Kodama T, Rokuda M, Park KS, Cantarelli VV, Matsuda S, Iida T, Honda T. 2007. Identification and characterization of VopT, a novel ADP-ribosyltransferase effector protein secreted via the *Vibrio* *parahaemolyticus* type III secretion system 2. Cell Microbiol 9:2598-2609.

5. Hiyoshi H, Kodama T, Iida T, Honda T. 2010. Contribution of *Vibrio* *parahaemolyticus* virulence factors to cytotoxicity, enterotoxicity, and lethality in mice. Infect Immun 78:1772-1780.

6. Ono T, Park KS, Ueta M, Iida T, Honda T. 2006. Identification of proteins secreted via *Vibrio* *parahaemolyticus* type III secretion system 1. Infect Immun 74:1032-1042.

7. Hiyoshi H, Kodama T, Saito K, Gotoh K, Matsuda S, Akeda Y, Honda T, Iida T. 2011. VopV, an F-actin-binding type III secretion effector, is required for *Vibrio* *parahaemolyticus*-induced enterotoxicity. Cell Host Microbe 10:401-409.
